# The Common Accidents of Every-Day Life

**Published:** 1887-10-01

**Authors:** Robert A. P. Forrester


### The Common Accidents of Every-day Life.

By Robert A. P. Forrester, B.A. Dub., M.B. and C.M.Edin.

### INFANCY.

"First the infant, mewling and puking in his nurse's arms."

*Foreign Bodies in the Pharynx and Œsophagus.*—It may be advisable, before considering the subject of foreign bodies in the pharynx and œsophagus, to give some idea of the arrangement of the parts. The pharynx is that part of the alimentary canal which receives the food after it has been masticated, and propels it down the gullet. It is a funnel-shaped muscular bag, about four inches and a half in length, with the broadest portion opposite the hyoid bone. The roof is attached to a portion of the occipital bone ; from this it descends perpendicularly as low as the cricoid cartilage in front and the fifth cervical vertebra behind, at which level the œsophagus begins. Its dimensions are not equal throughout. The breadth at the upper part is just equal to that of the posterior openings of the nose ; but it becomes wider where it transmits the food (*i.e.*, at the back of the mouth), thence it gradually contracts to the œsophagus. The pharynx may thus be compared to a funnel communicating in front by wide apertures with the nose, mouth, and larynx, while the œsophagus is the tube leading from its lower end.

The œsophagus begins where the pharynx ends, and passes through the diaphragm to the stomach, and is from nine to ten inches in length. Its course is not quite vertical, for in the neck it lies to the left of the windpipe, and in the chest it inclines to the right to make way for the aorta, and before passing through the diaphragm it again inclines to the left. In one part of its course it is in close relation with the pericardium for about two inches this may account for the pain experienced in swallowing food in pericarditis. In children allowance must be made for the smaller dimensions of the parts.

Young children very frequently introduce foreign bodies into their mouths, such as buttons, coins, pieces of food, bones, pins, needles, etc. The substance may become impacted, one very common situation being where the pharynx begins to contract towards the œsophagus. The involuntary efforts to swallow which are excited by the presence of the foreign body may only increase the evil, and unless relief be obtained speedily and effectually, suffocation may result from this accident. Should the foreign body, however, go beyond this point, it usually stops short at the termination of the œsophagus.

When the article is small and pointed, such as a bristle, pin, or needle, it may attach itself to the mucous membrane at the root of the tongue or sides of the pharynx. It may even penetrate into the larynx or through the œsophagus, and injure seriously the neighbouring parts. Great care is necessary, therefore, in the treatment of the vast majority of such accidents.

The symptoms in a young child who is unable to tell what is the matter with him, are merely objective, *i.e.*, such as may be observed by the surgeon himself, and are usually sufficiently evident. They are violent coughing, clutching a the throat perhaps, difficulty of breathing and swallowing. If the impaction be not relieved, suffocation may occur, or ulceration of the œsophagus, or an abscess may form, or some important blood-vessel may be penetrated and fatal hæmorrhage ensue.

Now what is the treatment to be in such a case? Clearly the removal of the foreign body. The *modus operandi*, however, must depend upon its nature and situation. The first thing to be done is to put the patient where a good light from a window or lamp can fall into the mouth, then carefully and quickly to examine the back of the pharynx and root of the tongue by passing the forefinger well down : search also between the pillars of the fauces and around the region of the hyoid bone. It is a good plan, too, to pull forward the tongue well out of the mouth, the organ being grasped with the fingers, covered with a handkerchief. Very possibly the offending substance may easily be grasped and hooked out with the finger—no forceps is so good as the finger and thumb of an operator who knows how to use them properly. If, however, it cannot be reached by the finger, then removal may be attempted by the forceps. Great care must be exercised in using the instrument, as matters may be made worse by the want of caution; the point of a sharp body may be driven further in, or a laceration made in the mucous membrane. When the substance is beyond the reach of the fingers or forceps, an expanding probang may be useful. This instrument consists of a fine whalebone stalk, tipped with sponge, ensheathed in a gum-elastic tube except for about two inches above the sponge. From the point where the sheath terminates strong bristles are arranged round the whalebone and attached above to the sheath and below to the sponge. After having introduced the instrument into the œsophagus we can expand the bristles in the manner of an umbrella by drawing back the whalebone stalk. On withdrawing it thus opened it sweeps the circumference of the gullet, and may catch any foreign body sticking in the wall. To introduce the instrument, oil it well, pass the fore finger of the left hand down the left side of the mouth, the head being thrown well back, and guide the probang vertically over the finger.

When the mass is rounded and smooth (*e.g.*, a piece of meat) and low down, it is a good plan to push it gently onwards by means of an ordinary sponge probang, introduced in the manner above described. But should the substance be rough, hard, and pointed, this method is not advisable, as perforation of the gullet might occur. What then is to be done in such a case? We must have recourse to the use of large forceps made specially for the purpose. If we fail with these, on account perhaps of the firm impaction, nothing remains but the performance of an operation for opening the œsophagus and extracting the body through the aperture made.

It is scarcely necessary to state that the above modes of treatment can only be carried out by a medical man. Before his arrival, however, something may be done by the friends of the patient to relieve him. It is of course permissible and right for any cool-headed person to attempt to dislodge the offending substance with the finger and thumb in the manner described. Should this fail on account of the mass (say a piece of meat) having gone out of reach down the gullet, where it may cause great discomfort, although not dangerous to life, the drinking of large draughts of water may cause it to pass on to the stomach. If not, the giving of an emetic will sometimes expel it by the vomiting produced. Common salt or mustard dissolved in water may be resorted to for this purpose.

*(To be continued.)*

